# Identification of cross-talk pathways and ferroptosis-related genes in periodontitis and type 2 diabetes mellitus by bioinformatics analysis and experimental validation

**DOI:** 10.3389/fimmu.2022.1015491

**Published:** 2022-09-29

**Authors:** Shengyuan Pan, Bo Hu, Jicheng Sun, Zun Yang, Wenliang Yu, Zangmin He, Xiang Gao, Jinlin Song

**Affiliations:** ^1^ College of Stomatology, Chongqing Medical University, Chongqing, China; ^2^ Chongqing Key Laboratory of Oral Diseases and Biomedical Sciences, Chongqing, China; ^3^ Chongqing Municipal Key Laboratory of Oral Biomedical Engineering of Higher Education, Chongqing, China

**Keywords:** ferroptosis, drug prediction, periodontitis, type 2 diabetes mellitus, pathway

## Abstract

**Purpose:**

There is a bidirectional relationship between periodontitis and type 2 diabetes mellitus (T2DM). The aim of this study was to further explore the pathogenesis of this comorbidity, screen out ferroptosis-related genes involved in the pathological process, and predict potential drug targets to develop new therapeutic strategies.

**Methods:**

Common cross-talk genes were identified from periodontitis datasets (GSE16134, GSE10334 and GSE106090) and T2DM databases (DisGeNET and GeneCard). Then, GO and KEGG enrichment analyses, PPI network analysis and hub gene identification were performed. The association between ferroptosis and periodontitis with T2DM was investigated by Pearson correlation analysis. Core ferroptosis-related cross-talk genes were identified and verified by qRT-PCR. Potential drugs targeting these core genes were predicted *via* DGIDB.

**Results:**

In total, 67 cross-talk genes and two main signalling pathways (immuno-inflammatory pathway and AGE-RAGE signalling pathway) were identified. Pearson correlation analysis indicated that ferroptosis served as a crucial target in the pathological mechanism and treatment of periodontitis with T2DM. IL-1β, IL-6, NFE2L2 and ALOX5 were identified as core ferroptosis-related genes and the qRT-PCR detection results were statistically different. In total, 13 potential drugs were screened out, among which, Echinacea and Ibudilast should be developed first.

**Conclusions:**

This study contributes to a deeper understanding of the common pathogenesis of periodontitis and T2DM and provides new insights into the role of ferroptosis in this comorbidity. In addition, two drugs with potential clinical application value were identified. The potential utility of these drugs requires further experimental investigation.

## Introduction

Periodontitis, triggered by microbiota dysbiosis, is an immuno-inflammatory disease of the periodontal tissue (1, 2). It affects approximately 61.9% of the worldwide population, making it one of the most significant lifestyle disorders (3). Type 2 diabetes mellitus (T2DM) is a metabolic disease characterised by hyperglycaemia, insulin resistance and insufficient insulin secretion (4, 5). In 2005, the International Diabetes Federation (IDF) reported that, worldwide, there were about 415 million people aged 20–79 years old living with T2DM, and it was estimated that this number will increase to more than 640 million by 2040 (6, 7). Both periodontitis and T2DM pose serious global public health and financial burdens, especially in developing economies (8–10).

In 2008, the American Diabetes Association (ADA) defined periodontitis as a complication of diabetes. Many epidemiological studies, clinical trials and scientific experiments have been performed in an attempt to elucidate the biological relevance of this relationship (11–13). Several epidemiological studies have provided strong evidence for a direct correlation between periodontitis and T2DM (14, 15). The risk of periodontitis in diabetics is 3-4 times that of patients without T2DM (16–18). Studies have also found increased expression of inflammatory factors in the gingival tissue of diabetic rats, including IL-1β, IL-6, TNF-α and IL-17A, which may be mediated by advanced glycation end products (AGEs) and their ligands (19, 20). On the other hand, periodontitis is not only a complication of diabetes but also a risk factor for it (11, 14). Several reports suggest that periodontitis can have an adverse effect on glycemic control in diabetes (12, 15). It is hypothesised that bacteria and their products in the periodontal pocket can enter the circulatory system through the damaged epithelial barrier, increasing the risk of insulin resistance and diabetic complications (11). Thus, today, scientists and doctors are increasingly using the term “comorbidity” to portray the bidirectional relationship between periodontitis and T2DM. Therefore, it is of great significance to study the interaction mechanism between periodontitis and T2DM to further understand the pathogenesis and treatment of these disorders.

Previous studies have provided preliminary evidence suggesting that the biological link between periodontitis and T2DM overlaps with the immuno-inflammatory response, microbiota dysbiosis and oxidative stress (11, 21, 22). There is growing literature indicating that an overabundance of reactive oxygen species (ROS) plays a role in the establishment of oxidative stress microenvironments that underlie the pathogenesis of many chronic inflammatory diseases, such as cardiovascular diseases, inflammatory bowel disease, periodontitis and T2DM (23–25). ROS can substantially increase the expression levels of proinflammatory factors, resulting in periodontal tissue destruction (26). As one of the main pathogenic factors in T2DM and its complications, the generation of AGEs is also related to ROS (27). Under normal conditions, ROS are beneficial for antimicrobial defence, signal transduction and gene regulation (28, 29). However, excessive ROS cause a series of pathological changes, mainly *via* lipid peroxidation; these changes include biological macromolecule and cell membrane damage, which is cytotoxic to the host cell and can even lead to cell death (30). Indeed, a variety of programmed cell death processes, such as apoptosis, pyroptosis, necroptosis and ferroptosis, are all related to the development of chronic inflammation (31, 32). Among them, ferroptosis is characterised by a dramatic increase in iron-dependent ROS, lipid peroxidation, a decrease in mitochondria and decreased or inactivated glutathione peroxidase-4 (GPX-4) activity; together, these lead to oxidative stress damage *in vivo* (33, 34). In the ferroptosis process, the cytoplasm may release a large number of danger signals, such as proinflammatory factors and iron, to the extracellular environment due to the rupture of the plasma membrane (23). Meng et al. reported that HMOX1 upregulation promotes ferroptosis in diabetic atherosclerosis (35). Thus, ferroptosis has become a target for study of the aetiology and treatment of various inflammatory diseases (33–35). Since ferroptosis and periodontitis with T2DM overlap biochemical pathways including ROS overload and lipid peroxidation, it is necessary to explore the role of ferroptosis in the pathogenesis of periodontitis with T2DM. However, to date, there is no published research on the role of ferroptosis in periodontitis with T2DM.

With the development of modern sequencing technology, bioinformatic analysis has allowed for the exploration of interrelationships and pathogenesis connections between diseases based on the use of human samples, rather than animal or cell models (36). This means that more convincing conclusions can be reached. Thus, using bioinformatics analysis and qRT-PCR validation, this study explored essential genes and signalling pathways in periodontitis and T2DM, provided new insights into the biological mechanisms of ferroptosis in periodontitis with T2DM and identified potential new therapeutic target drugs.

## Methods and materials

### Data collection

The GSE16134, GSE10334 and GSE106090 datasets were extracted from the Gene Expression Omnibus (GEO, https://www.ncbi.nlm.nih.gov/geo/) database. GEO is a public online database that is available to researchers. Each dataset contained both periodontitis samples and control samples. Text mining was performed on the DisGeNET and GeneCard databases to investigate genes related to T2DM.

### Identification of differentially expressed genes and cross-talk genes

After normalization and removal of the batch effect using R package inSilicoMerging, GEO2R (http://www.ncbi.nlm.nih.gov/geo/geo2r) was employed to identify DEGs between periodontitis and control samples. DEGs were identified based on P < 0.05 and |log fold change (FC)|≥ 0.5. The search term ‘type 2 diabetes mellitus’ was employed to identify and download all reported genes related to T2DM. To add authenticity, the genes overlapping in the two databases were identified as DEGs for T2DM. The common DEGs were identified as cross-talk genes of periodontitis and T2DM.

### Functional and pathway enrichment analysis of cross-talk genes

To comprehensively explore the functional information of these genes, Gene Ontology (GO) and Kyoto Encyclopedia of Genes and Genomes (KEGG) pathway enrichment analyses of the cross-talk genes were performed using DAVID (version 6.8, https://david.ncifcrf.gov/). GO enrichment analysis includes three parts: biological processes (BP), molecular functions (MF) and cellular components (CC). P < 0.05 and a false discovery rate (FDR) < 5% were the cut-off criteria.

### Protein-protein interaction network construction, hub gene identification and module analysis

To obtain a PPI network, the cross-talk genes were imported into STRING (http://www.string-db.org/), an online database used for predicting direct and indirect protein-protein functional interactions through correlation analyses. The combined score ≥ 0.4 was chosen for the PPI network construction. Then, the result was visualized using Cytoscape software (version 3.9.1). CytoHubba, a plugin in Cytoscape, was used to identify hub genes. Another plugin in Cytoscape, MCODE, was used to investigate the most significant module in the PPI network.

### Correlation analysis between cross-talk genes and ferroptosis-related genes

To explore the specific role of ferroptosis in periodontitis with T2DM, 388 FRGs were downloaded from the FerrDb website (http://www.zhounan.org/ferrdb/), including 186 drivers, 132 suppressors and 113 markers (**Supplementary Table S1**). The FerrDb website is an online database for identifying the newest genes related to ferroptosis. Then, the correlations between the cross-talk genes and FRGs were assessed using Pearson correlation analysis. The overlapping cross-talk genes and FRGs were defined as ferroptosis-related cross-talk genes (FR-cross-talk genes) for subsequent investigation. Functional and pathway enrichment analysis of FR-cross-talk genes was performed using the method described above.

### Receiver operating characteristic curve analysis

To evaluate the sensitivity and specificity of the FR-cross-talk genes for diagnosis, receiver operating characteristic (ROC) curve analysis was conducted using the R package pROC (version 1.17.0.1). The confidence interval was set as 95%. Genes with an area under the ROC curve (AUC) of more than 0.7 were considered significant and were defined as core genes (37, 38).

### Experimental validation of core genes by clinical samples

To experimentally validate the core genes, healthy gingival samples were harvested from six individuals without periodontitis or T2DM during crown lengthening surgery, and diseased periodontal samples in deep periodontal pocket were harvested from six individuals with periodontitis and four individuals with periodontitis and T2DM during periodontal flap surgery. Samples were immediately transferred to liquid nitrogen for storage. The diagnostic criteria for T2DM were those established by the ADA in 2021 (39), i.e., random plasma glucose ≥11.1 mmol/L or 2-h plasma glucose ≥11.1 mmol/L during Oral glucose tolerance tests (OGTT) or fasting plasma glucose ≥7.0 mmol/L or random plasma glucose ≥11.1 mmol/L or glycosylated hemoglobin A1C ≥6.5% (39). Periodontitis was diagnosed according to the 2017 World Workshop on the Classification of Periodontal and Peri-Implant Diseases and Conditions criteria (40). The exclusion criteria included the use of immunosuppressive drugs or antibiotics (for more than seven days within the past 30 days) or non-steroidal anti-inflammatory drugs (except daily use of aspirin 75-325 mg); the presence of systemic disease; and excessive alcohol use. All individuals were selected from the Affiliated Hospital of Stomatology, Chongqing Medical University and they had not had periodontal surgery before. Periodontitis or T2DM was diagnosed by qualified doctors. The study was approved by the Ethics Committee of the Affiliated Hospital of Stomatology, Chongqing Medical University (2022059) and complied with the 1964 Helsinki declaration and later amendments and ethics standards.

### Quantitative real-time polymerase chain reaction and statistical analysis

After sufficient grinding, total RNA was extracted from the gingiva using RNAiso Plus (Takara, Japan). Next, 5 × PrimeScript RT Master Mix (Takara, Japan) was used for reverse transcription and a TB Green PCR Core Kit (TaKaRa, Japan) was used for PCR amplification. The reaction and detection were conducted on a CFX96TM system (Bio-Rad, USA). Beta-actin was used as the standardized reference gene. The relative mRNA expression levels of each target gene were calculated using the 2-ΔΔCT method. The primer sequences of genes used in the study are listed in **Table 1**. The qRT-PCR data were analysed by ANOVA using GraphPad Prism software (version 6.0, USA). A p-value <0.05 was considered statistically significant. Radar chart was used to visualize different genes expression levels between groups.

**Table 1:** Primer sequences information used in this study.

Gene	Forward primer sequence (5′-3′)	Reverse primer sequence (5′-3′)
Beta-actin	AGAAAATCTGGCACCACACCT	GTGACGGGACCGTGGGTCGTG
IL-1β	TCTGTACCTGTCCTGCGTGT	ACTGGGCAGACTCAAATTCC
IL-6	GAGTAGTGAGGAACAAGCCAGAG	GGTCAGGGGTGGTTATTGC
NFE2L2	AGGTTGCCCACATTCCCAAA	ACGTAGCCGAAGAAACCTCA
ALOX5	CCGGCACTGACGACTACATC	TATGAATCCACCGCGCCAC

### Potential drugs prediction

The drug-gene interaction database (DGIDB, http://www.dgidb.org) is a free database that provides interaction information about drugs and genes of interest. In this study, DGIDB was used to screen potential drugs targeting core genes in order to identify new therapeutic targets. A query score >1 was set as the cut-off criterion (41, 42).

## Results

### Flowchart of the study and identification of cross-talk genes

**Figure 1:**
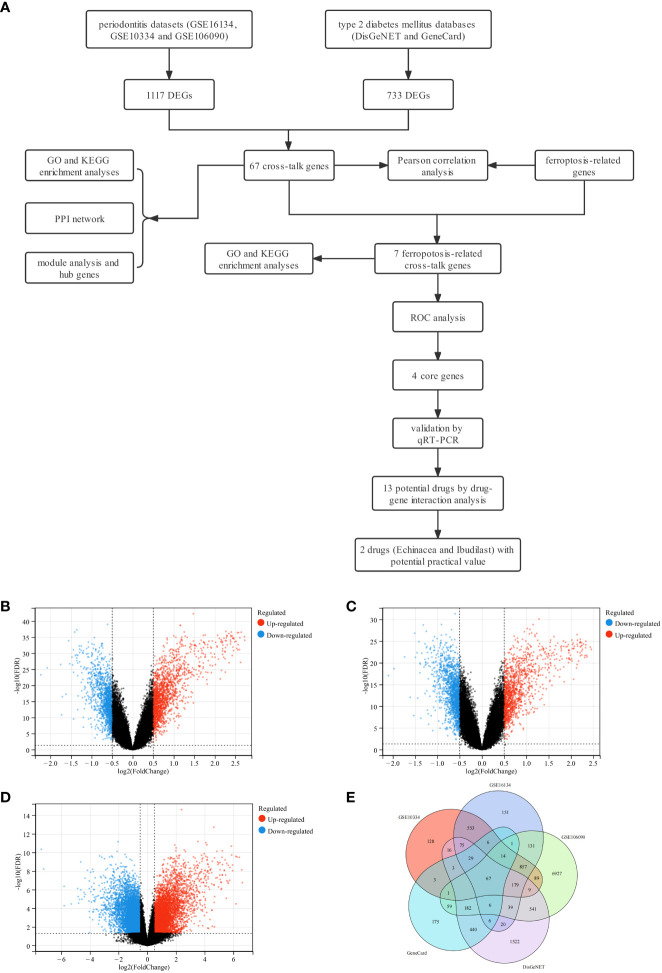
Study design flowchart, volcano diagram and venn diagram. **(A)** The detailed flowchart of the study. **(B)** The volcano map of GSE16134. **(C)** The volcano map of GSE10334. **(D)** The volcano map of GSE106090. Upregulated genes are marked in red; downregulated genes are marked in blue. **(E)** Venn diagram of cross-talk genes from three datasets and two databases.

A detailed flowchart of the study is presented in **Figure 1A**. In brief, a total of 1117 DEGs for periodontitis, 733 DEGs for T2DM and 67 cross-talk genes were identified through data mining and processing (**Figures 1B-E**). The genes were listed in **Supplementary Table S2**.

### Functional and pathway enrichment analysis of cross-talk genes

**Figure 2:**
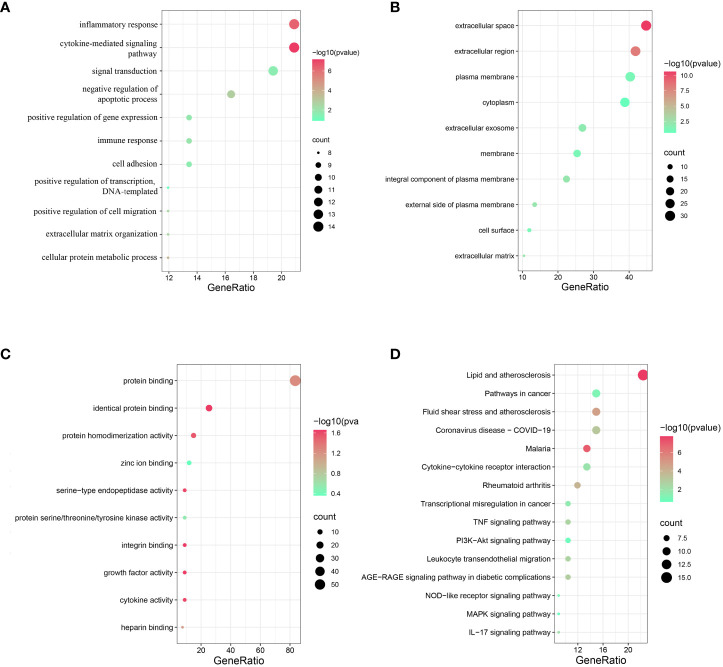
Functional and pathway enrichment analysis of cross-talk genes. **(A)** Top 10 BP terms. **(B)** Top 10 CC terms. **(C)** Top 10 MF terms. **(D)** Top 15 KEGG terms. P < 0.05 was considered significant.

For GO analysis, changes in BP included significant enrichment of several immuno-inflammatory processes, such as the cytokine-mediated signalling pathway, inflammatory response, signal transduction and immune response (**Figure 2A**). Changes in CC included significant enrichment of the basic structure of the cell, such as extracellular space, extracellular region, plasma membrane and cytoplasm (**Figure 2B**). Changes in MF included significant enrichment of binding-related functions, such as protein binding, identical protein binding, zinc ion binding and integrin binding (**Figure 2C**). For KEGG analysis, as expected, the cross-talk genes were enriched in several ferroptosis-related signalling pathways, such as the PI3K-Akt signalling pathway and MAPK signalling pathway; these are also two of the most prominent signalling pathways implicated in human inflammatory diseases (31–34). The cross-talk genes were also enriched in other pathways, including the AGE-RAGE signalling pathway in diabetic complications, TNF signalling pathway and NOD-like receptor signalling pathway (**Figure 2D**).

### PPI network, hub gene identification and module analysis

**Figure 3:**
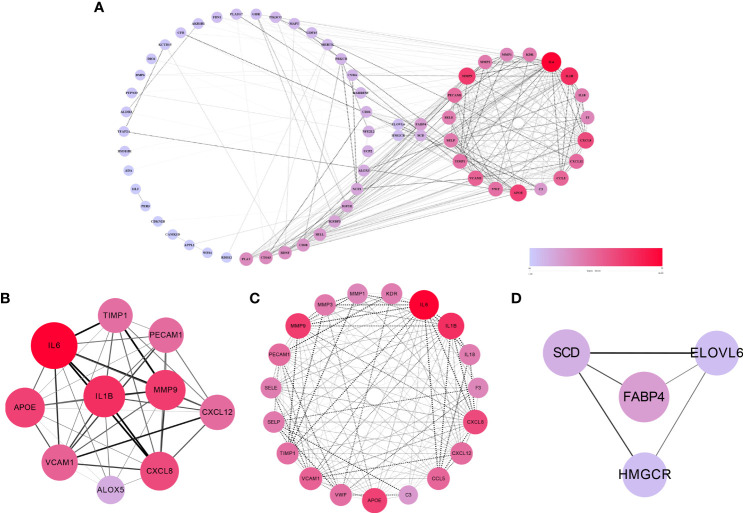
The results of PPI network, hub genes and significant modules. **(A)** PPI network of cross-talk genes. **(B)** Top 10 hub genes. **(C)** Significant module 1 (contained 19 nodes and 151 edges). **(D)** Significant module 2 (contained 4 nodes and 5 edges).

The 67 cross-talk genes from the above analysis were all uploaded to the STRING database to build a PPI network. The network contained 62 nodes and 336 edges (**Figure 3A**). Based on the MCODE scores, 10 hub genes (IL6, IL1B, MMP9, APOE, CXCL8, VCAM1, CCL5, CXCL12, PECAM1 and TIMP1) were screened out (**Figure 3B**). Next, the most significant modules were extracted, of which, module 1 contained 19 nodes and 151 edges (**Figure 3C**), and module 2 contained 4 nodes and 5 edges (**Figure 3D**). Both network and significant modules indicated that periodontitis and T2DM share some common potential molecular mechanisms.

### Correlation analysis between cross-talk genes and FRGs

**Figure 4:**
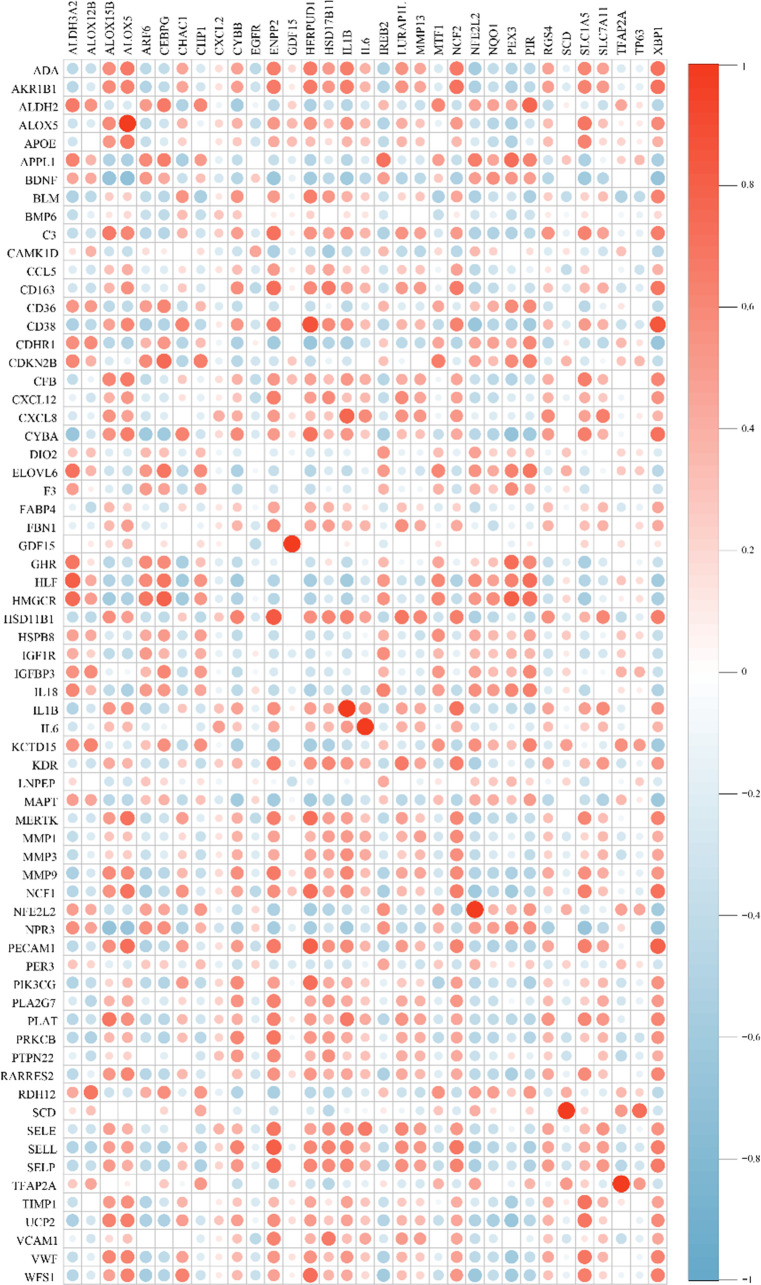
Correlation analysis between cross-talk genes and ferroptosis-related genes. Genes in the vertical line are cross-talk genes and genes in in the horizontal line are FRGs. Red indicates a positive correlation, blue indicates a negative correlation. The size of circle designates the correlation coefficient between cross-talk genes and ferroptosis-related genes.

**Figure 5:**
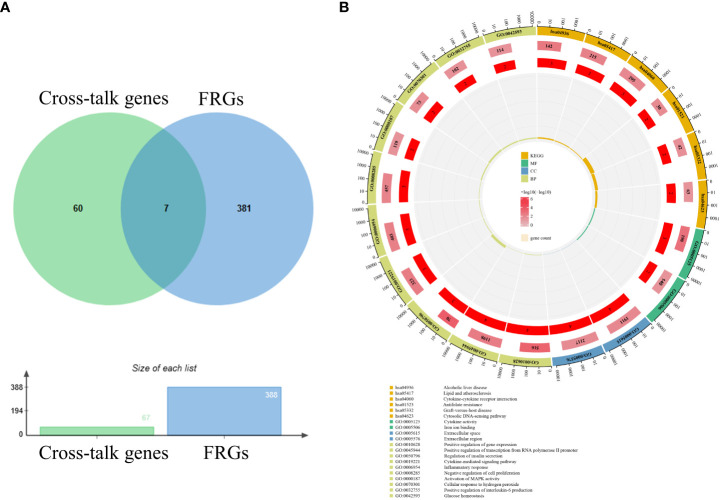
Venn diagram and enrichment analysis of FR-cross-talk genes. **(A)** Seven FR-cross-talk genes were identified by venn diagram. **(B)**The GO and KEGG enrichment analysis of FR-cross-talk genes.

After batch correction and normalization, a periodontitis dataset was obtained and correlation analysis between cross-talk genes and FRGs was carried out. The Pearson analysis results showed that all the correlation coefficients were statistically significant (P < 0.05) and most of the cross-talk genes had large correlation coefficient sums (**Figure 4**; **Supplementary Table S3**), indicating that ferroptosis may play a role in the pathological process of periodontitis with T2DM and may be a therapeutic target for future research. Then, a Venn diagram (**Figure 5A**) was used to screen out seven FR-cross-talk genes (IL-1β, IL-6, ALOX5, NFE2L2, GDF15, SCD and TFAP2A); the sums of their correlation coefficients were 29.71, 19.87, 30.09, 29.52, 8.77, 13.18 and 16.41, respectively (**Supplementary Table S3**).

### Functional and pathway enrichment analysis of FR-cross-talk genes

To explore the biological functions and pathways related to the FR-cross-talk genes, GO and KEGG analyses were performed (**Figure 5B**). The GO analysis results revealed that the FR-cross-talk genes were significantly enriched in i) regulation of insulin secretion, cytokine-mediated signalling pathway, inflammatory response, cellular response to hydrogen peroxide, positive regulation of interleukin-6 production and glucose homeostasis (BP); ii) extracellular space and extracellular region (CC); and iii) iron ion binding and cytokine activity (MF). The KEGG analysis results revealed that cytokine-cytokine receptor interaction, antifolate resistance and the cytosolic DNA-sensing pathway were significantly enriched. The above terms are related to iron metabolism, glucose metabolism, inflammation and oxidative stress, and some are consistent with the enrichment analysis results of the cross-talk genes.

### ROC curve analysis

**Figure 6:**
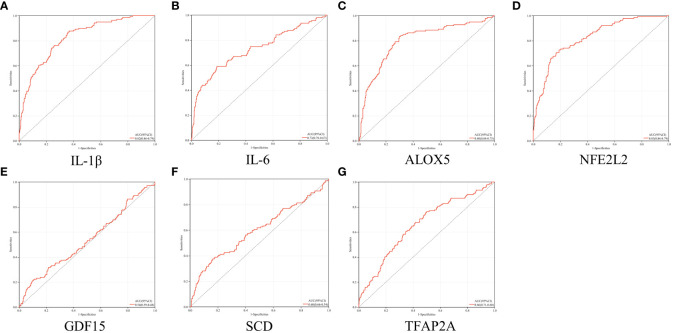
ROC analysis. (**A–G**) ROC analysis results of IL-1β, IL-6, ALOX5, NFE2L2, GDF15, SCD and TFAP2A, respectively.

As illustrated in **Figure 6**. the AUCs of IL-1β, IL-6, ALOX5 and NFE2L2 were 0.82, 0.73, 0.80 and 0.83, respectively. The four genes were considered to be of high accuracy in diagnosing periodontitis with T2DM and were defined as core genes (**Supplementary Table S4**). On the contrary, GDF15, SCD and TFAP2A had lower sensitivity in predicting periodontitis with T2DM, with AUCs of 0.54, 0.60 and 0.66, respectively.

### Validation of core gene expression by qRT-PCR

**Figure 7:**
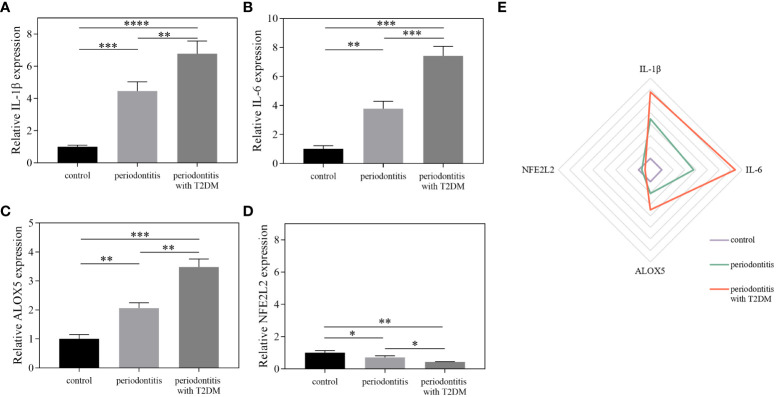
The results of qRT-PCR. **(A–D)** The relative expression levels of IL-1β, IL-6, ALOX5 and NFE2L2 between different groups using qRT-PCR. **(E)** Differentially expressed factors were shown as a Radar Chart. The qRT-PCR data were analysed by ANOVA. A p-value < 0.05 was considered statistically significant. All data were presented as the mean ± SD. *P < 0.05, **P < 0.01, ***P < 0.001, ****P < 0.0001.

To further increase our confidence in the findings, qRT-PCR was used to detect the expression of the core genes in the different sample groups (**Figure 7**; **Supplementary Table S5**). Compared with the control group, the mRNA expression levels of IL-1β, IL-6 and ALOX5 were increased in the periodontitis group and further increased in the periodontitis with T2DM group. The expression levels of IL-1β and IL-6 were higher than the other two genes in the periodontitis group and periodontitis with T2DM group. The mRNA expression level of NFE2L2 was decreased in the periodontitis group and further decreased in the periodontitis with T2DM group. The results indicate that periodontitis can lead to inflammatory and oxidative stress damage, and that T2DM can play a synergistic role.

### Drug-gene interaction analysis

To predict drugs with potential therapeutic effects for periodontitis with T2DM, the five core genes were introduced into DGIDB. In total, 37, 25, 821 and 25 target drugs were obtained for IL-1β, IL-6, NFE2L2 and ALOX5, respectively (**Supplementary Table S6**). Most of the drugs were anticancer, inhibitor, antibody or antagonist drugs. Drugs that target more than one gene were considered more credible and effective (**Table 2**). Some of them, including Methotrexate, Melatonin, Resveratrol, Diacerein, Lithium and Infliximab, have been used to treat periodontitis or T2DM in animal experiments (43–47, 49), while the remaining drugs (Hydroquinone, Echinacea, Hydrocortisone, Cisplatin, Omeprazole, Ibudilast, Lansoprazole) have not been found to have therapeutic effects in periodontitis with T2DM. Notably, Cisplatin, a metal-based anticancer agent, has been reported to aggravate periodontitis (48).

**Table 2:** Drug-gene interaction analysis and literature search.

Drug	Targeted genes	Reported in previous studies (Yes or No)	Reference
Methotrexate	ALOX5, NFE2L2	Yes	Lübcke P.M. et al., 2019 (43)
Melatonin	ALOX5, IL-1β	Yes	Kose O. et al., 2021 (44)
Hydroquinone	IL-1β, NFE2L2	No	/
Resveratrol	IL-1β, NFE2L2	Yes	Bhattarai G. et al., 2016 (45)
Echinacea	IL-1β,IL-6	No	/
Diacerein	IL-1β, NFE2L2	Yes	Silva R.C.L. et al., 2022 (46)
Lithium	IL-1β, NFE2L2	Yes	de Souza Malta F. et al., 2020 (47)
Hydrocortisone	IL-1β, NFE2L2	No	/
Cisplatin	IL-6, NFE2L2	Yes	Gusman D.J.R. et al., 2019 (48)
Omeprazole	IL-1β, NFE2L2	No	/
Ibudilast	IL-1β,IL-6	No	/
Infliximab	IL-1β,IL-6	Yes	Kim J.H. et al., 2017 (49)
Lansoprazole	IL-1β, NFE2L2	No	/

## Discussion

Periodontitis and T2DM are global epidemic diseases that involve multiple genes and signalling pathways (1, 5). Clinically, periodontitis and T2DM are risk factors for each other, showing a bidirectional link, but the underlying mechanisms responsible for the link are not fully understood. Oxidative stress plays an important role in the pathogenesis and therapeutic targets of periodontitis and T2DM (22, 50, 51). Moreover, ferroptosis can cause oxidative damage and oxidative stress imbalance *in vivo*. At present, no study has examined the role of ferroptosis in periodontitis with T2DM. Thus, in this study, we attempted to investigate the association and mechanism between the two diseases, explore the role of ferroptosis-related genes in periodontitis with T2DM, and search for potential effective therapeutic target drugs using bioinformatics analysis combined with experimental validation.

In the present study, 67 cross-talk genes that were overlapping in periodontitis and T2DM were identified, including 10 hub genes (IL-6, IL-1B, MMP9, APOE, CXCL8, VCAM1, CCL5, CXCL12, PECAM1 and TIMP1). Functional and pathway enrichment analyses demonstrated that these cross-talk genes were mainly enriched in immuno-inflammatory pathways, such as cytokine-cytokine receptor interaction, TNF signalling pathway, PI3K-Akt signalling pathway, leukocyte transendothelial migration, IL-17 signalling pathway, immune response and cytokine activity. At present, periodontitis and T2DM are considered to be chronic inflammatory diseases characterized by the production of a large number of proinflammatory factors (11, 20, 22). In periodontitis, increased expression levels of proinflammatory factors stimulate osteoclast activity by altering the RANKL/osteoproteinin (OPG) ratio, leading to progressive alveolar bone resorption (52). At the same time, periodontitis contributes to low-grade systemic inflammation by remote transfer of local bacteria, bacterial products and proinflammatory mediators (21), which can worsen insulin resistance and dysglycemia, fuelling the development of T2DM (11). On the other hand, T2DM upregulates the expression of proinflammatory factors in local periodontal tissues and the circulatory system (22, 51). Higher levels of IL-17, PGE2, IL-23 and interferon-γ have been found in the gingival crevicular fluid (GCF) of patients with periodontitis with T2DM, compared to systematically healthy patients with periodontitis (19, 20). Importantly, the above processes interact and form a vicious cycle (11, 53, 54). According to the KEGG results of this study, the AGE-RAGE signalling pathway is also closely associated with both periodontitis and T2DM. AGEs are formed by irreversible glycosylation and oxidation of proteins, nucleic acids and lipids caused by hyperglycemia (55). The combination of AGEs and receptor of AGEs (RAGEs) increases the expression of proinflammatory cytokines (such as IL-6, TNF-α, IL-1β) to promote periodontal inflammation (55, 56). With the activation of RAGEs and other receptors, nuclear factor-Kappa B is also activated, which leads to an increase in ROS and oxidative stress damage (56). The accumulation of AGEs in alveolar bone interferes with bone remodelling and regeneration by disrupting the normal physiological function of the RANKL/OPG axis (57).

It is well-accepted that oxidative stress plays a critical role in the pathophysiological process of periodontitis and T2DM, whereas ferroptosis is closely related to the establishment of an oxidative stress microenvironment characterized by ROS production and lipid peroxidation in inflammatory diseases (23–25). FRGs may play a driving, suppressing and/or marking role in ferroptosis (58). Cross-talk genes are common DEGs in periodontitis and T2DM. The GO and KEGG enrichment analyses illustrated that FRGs and cross-talk genes jointly regulate the biological processes related to oxidative stress, iron metabolism, glucose metabolism and inflammation, suggesting a potential association between ferroptosis and periodontitis with T2DM. Additionally, we further investigated the link between ferroptosis and comorbid periodontitis and T2DM by performing Pearson correlation analysis between the FRGs and cross-talk genes. The results showed that most of the cross-talk genes were highly correlated with the FRGs, indicating that ferroptosis is an important target for studying the aetiology and treatment of periodontitis with T2DM. Then, we identified four core genes (IL-1β, IL-6, NFE2L2 and ALOX5) from the ferroptosis-related cross-talk genes by ROC curve analysis. The AUCs of these four genes were all greater than 0.7, suggesting that they have clinical value for diagnosing and monitoring periodontitis with T2DM. Furthermore, we collected clinical samples from different patient populations (control group, periodontitis group and periodontitis with T2DM group) and conducted qRT-PCR to verify the above results. The qRT-PCR results revealed upregulation of IL-1β, IL-6 and ALOX5 in the periodontitis group and further upregulation in the periodontitis with T2DM group, while the expression of NFE2L2 was the opposite. This is consistent with the ROC analysis results. Both IL-1β and IL-6 are crucial mediators of the inflammatory response, and the expression levels of IL-1β and IL-6 can be downregulated by inhibiting ferroptosis (59). Nuclear factor erythroid-derived 2-like 2 (NFE2L2), also known as Nrf2, encodes a transcription factor that binds to protective genes with antioxidant elements in the promoter region to rescue oxidative damage (58). Cui et al. reported that RSL3 induces Nrf2 expression to protect cells from ferroptosis in lipopolysaccharide-induced inflammation (60). Arachidonate 5-lipoxygenase (ALOX5) encodes a protein that is a member of the lipid oxidase family and catalyses the oxidation of arachidonic acid to produce leukotriene (61). According to previous studies, ALOX5 plays a crucial role in cell death, including apoptosis, pyroptosis, necroptosis and ferroptosis. Upregulation of ALOX5 induces ferroptosis through the accumulation of leukotriene and lipid peroxidation, which exacerbates the inflammatory response (61, 62).

Based on the results, 13 drugs with effective therapeutic effects or priority for development were screened out. Aside from Cisplatin, which aggravates periodontitis, Methotrexate, Melatonin, Resveratrol, Diacerein, Lithium and Infliximab have been reported to have therapeutic effects on periodontitis or T2DM *via* different pathways in animal models (43–49). The remaining six drugs (Hydroquinone, Echinacea, Hydrocortisone, Omeprazole, Ibudilast and Lansoprazole) have not been reported in the treatment of periodontitis with T2DM. Among them, Echinacea and Ibudilast deserve our attention. Echinacea, a herb belonging to the family Asteraceae, is a traditional medicine with antibacterial, antioxidant and immunomodulatory activities (63). Its main chemical components are polysaccharides, volatile terpenes, caffeic acid derivatives, alkylamides, polyphenols and alkaloids, and these components may regulate the MAPK signalling pathway, JNK signalling pathway and Nrf2/HO-1 signalling pathway (64). Because of its excellent pharmacological activities, Echinacea has been used in the clinical treatment of the common cold, respiratory infections, conjunctivitis and some cancers, and has been shown to be safe (63, 64). Ibudilast is a safe orally-available phosphodiesterase 4 inhibitor that has been used in clinical practice for over 20 years. It inhibits the production of proinflammatory factors and the Th17 response *in vivo* to exert anti-inflammatory effects (65, 66). Ibudilast can be used to treat acute kidney injury and rheumatoid arthritis in animal models (67). These drugs are potential new target drugs for the treatment of periodontitis with T2DM; however, more animal model and clinical cohort studies will be needed to verify their use.

Although previous studies have separately explored the role of ferroptosis in periodontitis and T2DM through bioinformatics analysis (68–70), few studies have reported the potential relationship between ferroptosis and periodontitis with T2DM. This study might provide new insights into the biological mechanisms of ferroptosis in periodontitis with T2DM. Moreover, potential targeted drugs were predicted. However, there are some limitations to the present study. Firstly, since this study is based on existing public datasets, the results are largely exploratory and should be treated with caution. Secondly, the FRGs are derived from FerrDb, a continuously updated website, and more related genes are yet to be discovered. Moreover, the four ferroptosis-related core genes (IL-1β, IL-6, NFE2L2 and ALOX5) identified in this study are also involved in other pathways. Therefore, future studies are needed to explore the specific role of these four genes in ferroptosis.

## Conclusion

In conclusion, through bioinformatics analysis and qRT-PCR validation, we identified common hub genes and signalling pathways involved in periodontitis and T2DM and demonstrated a strong association between ferroptosis and this comorbidity. In addition, four ferroptosis-related core genes (IL-1β, IL-6, NFE2L2 and ALOX5) were identified and verified by qRT-PCR. These may serve as potential biomarkers for the clinical diagnosis and treatment of periodontitis with T2DM. Meanwhile, 13 drugs targeting these core genes were screened, among which, Echinacea and Ibudilast appear to be of potential practical value in the treatment of periodontitis with T2DM. This study provides novel insights into the pathogenesis and treatment of periodontitis with T2DM. The findings of this study should be validated in future clinical and experimental studies.

## Data availability statement

The original contributions presented in the study are included in the article/**Supplementary Material**. Further inquiries can be directed to the corresponding authors.

## Ethics statement

The studies involving human participants were reviewed and approved by Ethics Committee of the College of Stomatology, Chongqing Medical University (2022059). The patients/participants provided their written informed consent to participate in this study.

## Author contributions

JSong and SP developed and designed the major study plan. SP and BH analyzed the data, interpreted the data and draw charts. JSun and ZY collected the literature and reference. ZH and WY helped checked the data. SP wrote the first draft of the paper. JSong and XG supervised the study. All authors contributed to the article and approved the submitted version.

## Funding

This study was supported by the National Natural Science Foundation of China (31600788), the Chongqing Research Program of Basic Research and Frontier Technology (cstc2015jcyjA10035), the Postgraduate Students’ Innovative Research and Development Projects of Chongqing (CYB19158), 2019 Chongqing Graduate Tutor Team Construction Project (dstd201903).

## Conflict of interest

The authors declare that the research was conducted in the absence of any commercial or financial relationships that could be construed as a potential conflict of interest.

## Publisher’s note

All claims expressed in this article are solely those of the authors and do not necessarily represent those of their affiliated organizations, or those of the publisher, the editors and the reviewers. Any product that may be evaluated in this article, or claim that may be made by its manufacturer, is not guaranteed or endorsed by the publisher.
